# The frequency of human papillomavirus findings in normal oral mucosa of healthy people by PCR

**DOI:** 10.1590/S1808-86942010000100013

**Published:** 2015-10-17

**Authors:** David Esquenazi, Ivo Bussoloti Filho, Maria da Gloria da Costa Carvalho, Fernando Souza de Barros

**Affiliations:** 1 MSc in Medicine (otorhinolaryngology), Federal University of Rio de Janeiro; PhD in Otorhinolaryngology, Santa Casa de São Paulo Medical Sciences School. Assisting Professor, Medical School, Federal University of Rio de Janeiro; 2 PhD in Medicine (otorhinolaryngology), University of São Paulo. Adjunct Professor, Santa Casa de São Paulo Medical Sciences School; 3 Post-PhD, National Institutes of Health (United States). Head of the Laboratory - CCF Biophysics Institute, UFRJ, Brazil; 4 4th year Medical Student, UFRJ Medical School. Student at the Scientific Initiation Program, UFRJ Medical School

**Keywords:** mouth, papillomavirus infections, mouth mucosa

## Abstract

The human papillomavirus (HPV) is a DNA virus, which belongs to papillomaviridae family, being of low and high risk, which infect the skin and mucous membranes and can induce benign and malign tumor formation. In the oral mucosa they have been associated with oral papilloma, focal epithelial hyperplasia, leucoplakia and oral neoplasia.

**Aim:**

to study the frequency of HPV finding in oral mucosa of normal people.

**Materials and methods:**

Prospective study, cross-sectional cohort. One hundred volunteers, young adults, healthy, aged between 20 and 31 years, university students with no history, no complains, without oral or oropharyngeal lesions. They were submitted to a questionnaire with questions regarding HPV infection epidemiology. The samples were harvested by brushing and analyzed by PCR.

**Results:**

The results were negative for HPV in all samples.

**Conclusion:**

It seems we had high social and economical class individuals, with nutrition rich in carotenoyds and vitamin C, low smoking and alcohol consumption and heterosexual habits with predominant monogamy and regular use of condoms.

## INTRODUCTION

Skin and mucosa are the preferential sites infected by the human papillomavirus. HPV is frequently associated with benign and malignant neoplasms in the oral cavity, the most commonly of which being squamous cell carcinoma. The presence of HPV in the epithelium of normal oral mucosae, as reported in the literature, does not allow for accurate inferences regarding the virus' role on carcinogenesis, as to whether it is the main etiologic agent, merely a secondary force, or simply another inhabitant of the oral epithelium. In spite of the improvements introduced to oral mucosa HPV detection techniques, the virus direct involvement in the appearance of oral carcinomas is yet to be definitively proven^1^. However, considering the HPV prevalence rates seen in lesions associated with the virus^2^, its participation in carcinogenesis cannot be ruled out.

The human papillomavirus (HPV) belongs to the papillomaviridae genus, papovaviridae family, and has a double circular DNA strand with approximately 8,000 base pairs^3^. This is a small non-enveloped virus, and its genome has its genes coded. Its capsid is icosahedral with a diameter ranging between 50nμ and 60nμ; the virus is not coated by a lipid envelope; it has 72 capsomeres and species-specific antigenic determiners in its outer surface and internally. This virus has a double DNA strand divided into early (E) and late (L) proteins, and regulating regions. The early region (E1-E8) codes genes for viral replication and host cell transformation. Proteins E6 and E7 are coded for oncogenic HPV types and have been shown to have a role in negatively affecting the cell cycle by binding and deactivating tumor suppressing genes p53 and pRB. The late regions code capsid proteins L1 and L2. L1 is the largest capsid and it is highly preserved among the HPV types. The long control region (LCR) is a regulating region that contains the origin of viral replication that occurs exclusively in the nucleus of the host cell1,^4^, ^5^, ^6^, ^7^. Depending on the composition of these segments, various types of HPV are identified with different pathogenic characteristics. Benign oral lesions are associated with low-risk HPV types 2, 4, 6, 7, 11, 13, and 32, with a low potential for evolving to malignancy. Malignant oral lesions are associated with high-risk HPV types 16, 18, 31, 33, 35, 39, 42, 45, 51, 52, 56, 58, 59 and 66.^8^, ^9^, ^10^, ^11^ Given the presence of HPV in normal oral mucosae in the form of latent infection, the strong evidences indicating that benign HPV lesions may evolve aggressively and participate in carcinogenesis, the limited and often contradictory knowledge on viral behavior on normal and diseased individuals, and the evidences towards the increased risk of developing malignant lesions among persons infected with high-risk HPV, it is utterly important that HPV in the macroscopically normal oral mucosa of healthy individuals be researched.

HPV cannot infect us through an intact squamous epithelium. In vivo, it infects the basal layer of mitotically active skin or mucosa through abrasions or wounds inflicted upon the epithelium.^12^ It is disseminated by direct cell-to-cell contact without the classical signs of viremia.^13^ After it penetrates the cell, the viral genome is transported to the cell nucleus where it is translated and transcribed. Viral genome is replicated in the following stages: first, early proteins (E1 and E2 are synthesized. As a result of that, some 10 to 200 genome copies are replicated per cell. In the second stage, during the cell cycle, replication occurs in offspring cells at an equal rate. Expression of genes E6 and E7 leads to cell transformation or differentiation. Cells start presenting a faster life cycle and begin to divide more frequently, leading to the formation of benign tumors. At this point, the virus proliferates in the tissue without destroying the cell hosting it. On the third stage, also referred to as the productive stage, large amounts of proteins E1 and E2 start producing thousands of copies of viral deoxyribonucleic acid (vDNA). On the other hand, late proteins (L1 and L2) - fundamental for new virus assembly - are also produced. Viruses are then released from the keratocyte located more superficially.^6^,^12^ In infections by high-risk strains, viral proteins E6 and E7 - a.k.a. oncoproteins - are highly active and interfere deeply with the cell cycle. This results in faster cell division when compared to infections by lower risk strains, thus increasing the probability of vDNA being integrated to cell genome. Such integration seems to be the cause of carcinogenesis.^4^, ^5^, ^6^,^10^

Sexual intercourse is the main mode of transmission of HPV.^14^ When considering transmission to the oral cavity, there seems to be a maternal-fetal pathway, while other mechanisms may occur after the neonatal period such as inoculation through skin lesions onto another individual or self-inoculation^15^,^16^. Some authors have considered that in adults the main pathway for oral HPV infection is oral sex^17^, but the transmission mechanism from the genital tract to the oral mucosa and vice-versa is yet to be completely clarified. Others consider that genital HPV infection is the most frequently observed viral disease among the more sexually active portion of the population.18 Studies on the concurrent presence of genital and oral HPV found the virus on normal mucosal tissue of women with genital HPV infection through Southern Blot hybridization (SBH - 15.6% - 33/212) and through polymerase chain reaction (PCR - 23.1% - 18/78) positive for oral HPV types 6, 11, 16, and 18.^19^ No positive results were found for oral HPV from samples scraped off of the normal oral mucosae of 30 patients with gynecologic HPV confirmed through PCR2; males (n=27) with anogenital lesions and without oral lesions had no HPV in their oral mucosae. This same study reported that in a group of three individuals with concurrent anal, genital and oral lesions only one was positive for HPV.^13^

In terms of carcinogenesis, in situ hybridization was performed only to find HPV types 16 and 18 in oral cavity squamous cell carcinomas.^20^ The authors of a literature review on oral carcinogenesis found evidences that HPV types 16 and 18 are associated with early proteins that bind, sequester, and degrade tumor suppressing genes, with E6 acting on p53 and E7 similarly upon pRB1. They also observed that vDNA was integrated into tumor cells after division, indicating their participation in oncogenesis.^21^,^22^

Authors looking at changes in eating habits indicated that regular intake of papaya, a fruit rich in carotenoids (lutein, zeaxanthin, and β-cryptoxanthin), and citric fruits (vitamin C) led to a reduction on the persistence of infection by HPV^23^; evidences also indicate that increased levels of folic acid reduce the risk of infection for high-risk HPV strains.^24^

According to the literature, prevalence rates of HPV in normal oral mucosae vary substantially. Our survey found values ranging from 0% to 100%19,25–36, as seen on Table 1.

**Table 1:** HPV in normal oral mucosa of healthy individuals (literature).

AUTHOR year	Age Range	Collected Material	Collection Method	Test	Total #	HPV+	total	Tipos de HPV
Maitland et al, 1987^25^	adults	total	biopsy	hibridizaçâo	12	5	41,6%	16
Scully et al., 1987^26^	adults	Oral mucosa	biopsy	hibridizaçâo	12	4	41,0%	16
Jenison et al., 1990^27^	adults	Oral mucosa	scrapes	PCR	35	11	31%	6,16
Yeudall, Campo, 1991^28^	adults	Oral mucosa	biopsy	PCR	25	2	8%	18
Jalal et al., 1992^29^	adults	oral mucosa	scrapes	PCR	48	25	52%	16
Kellokoski et al., 1992^19^	adults	Oral mucosa	biopsy	PCR	78	18	23,1%	6,11,16,18
				SB	212	33	15,6%	6,11,16,18
Cruz et al., 1996^30^	adults	normal gingiva	biopsy	PCR	12	0	0%	
Badaracco et al., 1998^31^	adults	Oral mucosa	scrapes	PCR	22	4	18%	6,11,16,31
Schwartz et al, 1998^32^	adults	Oral mucosa	scrapes	PCR	435	39	9%	6,11,16,18,31,33,35
Smith et al., 1998^33^	adults	Oral mucosa	lavage	PCR	205	10	4,80%	12,16,23,38 58,72 e nao classificado
Terai et al., 1999^34^	adults	Oral mucosa	scrapes	PCR	30	30	100%	6,16,18,59 61
Sand et al., 2000^35^	adults	Oral mucosa	biopsy	PCR	12	0	0%	-
Smith et al., 2004^36^	adults	Oral mucosa	lavage	PCR	333	61	10,80%	16,18,31,58

HPV=human papillomavirus

PCR=polymerase chain reaction; SB=Southern Blot hybridization

## OBJECTIVE

This paper aims to enhance the knowledge on HPV epidemiology and prevalence rates in healthy individuals, so that future studies can determine which protection factors are present in these individuals, which mechanisms are in effect and how the virus participates in the genesis of tumors, so prophylactic and therapeutic measures are developed for the benefit of larger populations.

## MATERIALS AND METHOD

This prospective cross-sectional survey was conducted between May 17, 2006 and April 11, 2007. One-hundred individuals with ages ranging between 20 and 31 years volunteered to participate in this study. Only individuals without previous history, complaints or present macroscopic oral cavity and oropharyngeal lesions were enrolled. All individuals answered a standard unidentified questionnaire numbered based on the collected samples. Epidemiologic factors connected to the transmission and persistence of HPV infection were addressed, such as gender, ethnicity, smoking, alcoholism, use of illicit drugs, sexual habits, number of sex partners, use of condoms, and eating habits specifically related to foods rich in vitamin C (citric fruit) and carotenoids (papaya). After carefully examining the individuals oral cavity and oropharynx using a photophore, samples were collected from the oral mucosa and the mastication sites using an Endobrush® brush. Samples were kept in refrigerated sterilized sealed numbered packages and sent to the Genic Expression Laboratory at the Carlos Chagas Filho Biophysics Institute to be analyzed through PCR (polymerase chain reaction). PCR was done using primers GP5+/GP6+ and MY09/ MY11 added to the DNA samples along with enzyme Taq polymerase, into the reaction compound containing all other components required for DNA synthesis. Then the reaction compound was placed on a thermocycler and submitted to repeated amplification cycles at different temperatures following the reaction phases: denaturation at 94°C, hybridization at 60°C, and synthesis at 72°C. The final product was amplified and analyzed through polyacrylamide gel electrophoresis. All reactions included a negative control (containing all components except for the DNA) and a positive control (in which a known standard gene was amplified).

This study was approved by the Research Ethics Committee at the Clementino Braga Filho University Hospital and registered before the Scientific Research Committee under the following permits: CEP:090/06 and CIC:062/06.

## RESULTS

Questionnaire results - sample characterization: Forty of the 100 individuals enrolled in our study were males and 60 were females; 86 claimed to be Caucasian, nine were brown and five were black; ages ranged between 20 and 31 years with a mean value of 22.65 years. In terms of tobacco consumption, 97/100 were non-smokers and 3/100 were smokers. Alcohol drinking was described in the following manner: 31/100 claimed not to have any alcohol; 56/100 have alcohol once a week; 3/100 have alcohol twice a week; 6/100 have it three times a week; 4/100 did not respond. Sex habits, as reported: 100/100 claimed to be heterosexuals; 85/100 have only one sex partner; 13/100 have more than one partner; 69/100 have oral sex; 22/100 do not have sex; 9/100 did not respond; 46/100 always wear condoms; 26/100 use condoms sometimes; 20/100 never wear condoms; 8 did not respond. Consumption of citric foods (vitamin C): 47/100 have them once a week; 26/100 have citric foods twice a week; 15/100 three times a week; 2/100 did not respond. Papaya consumption (carotenoids): 29/100 have it once a week; 5/100 twice a week; 5/100 three times a week; 53/100 do not have it; 8/100 did not respond.

### PCR results:

The questionnaire results on sample characterization can be seen on Table 2. PCR results were negative for all individuals involved.

## DISCUSSION

Social-economic factors: anogenital infections by HPV appear to be directly connected to social and economic variables.^14,^^35,^^36^ Authors have reported that virgin adolescents usually do not have detectable levels of anal or genital HPV, while those sexually active from earlier ages and individuals engaged in prostitution are more prone to having sexually transmitted diseases, HPV infection included. Low levels of HPV infection seem to be related to higher social and economic standards observed in the European population.^37^ In our sample, college students with average socio-economic status had negative PCR test results.

Eating habits: regular consumption of carotenoids (lutein, zeaxanthin, and β-cryptoxanthin) and vitamin C suggested reduced persistence of HPV infection^23^ and evidences indicate that increased levels of folic acid are inversely related to high-risk HPV infection^24^. In our sample, consumption of nutrients (vitamin C and carotenoids) appeared at a significant rate, as 88% (88/100) claimed to have citric foods (sources of vitamin C) between once and three times a week and 39% (39/100) stated they have papaya (source of carotenoids) between once and three times a week. These factors might have contributed to reduced prevalence rates of HPV in our sample.

Sex habits: all subjects in our sample claimed to be heterosexuals (100%, 100/100). Considering that having multiple partners is a factor more frequently found among homosexuals, one may assume that heterosexuality has contributed to the reduction on the virus prevalence rates in our sample; however, no specific studies on HPV prevalence rates among homosexuals were found. In our sample individuals were mostly monogamists, as 6% (6/100) claimed to have less than one partner per week, 85% (85/100) only one partner per week, 5% (5/100) two to three partners per week, 2% (2/100) more than three partners per week, and 2%(2/100) did not respond. This information is reinforced by our results, as 92% (92/100) of the subjects claimed to have only one partner per week when it comes to kissing the lips of another person. It is known that multiple contacts on one same day is rather common among young people in our society (thus the question was included in the questionnaire). Additionally, we must consider the relevance of the reported use of condoms; 46% (46/100) claimed they always use it, 26% (26/100) use it sometimes. The few individuals - 3% (3/100) - who reported to have sex with three partners per week and claimed not to wear condoms 1% (1/100) or to wear them sometimes - 2% (2/100), also had negative test results for HPV. The predominantly monogamist behavior and of using condoms by the subjects in our sample may have contributed for the absence of HPV positive test results. Although 69% (69/100) of the subjects in our sample stated they had frequent oral sex, considered by some as the principal infection pathway for oral HPV17, many authors believe there is no clear or proven link between oral sex and greater predisposition to acquiring oral HPV infection^7^,^13^.

Impact of tobacco and alcohol: differently from what was observed in genital cancer^38^, the impact of smoking in the progress of high-risk HPV infection and oral and oropharyngeal cancer is more controversial, sometimes dissociating such factors^39^, at times seeing increases^40^ and even decreases^41^, ^42^, ^43^ in tumor prevalence rates. Although some reports indicate that tobacco and alcohol may be risk factors for oral and oropharyngeal carcinoma, increased incidence of SCC in populations where tobacco and alcohol consumption were reduced for some time indicates other risk factors might be at play^43^. Reinforcing this idea, authors reported on male patients with oral and oropharyngeal SCC in which the group of heavy smokers had lower rates of infection by HPV^44^.

There seems to be a preventive effect of tobacco in oral and oropharyngeal infection by HPV, resulting probably from increases in keratinization of mucosal surfaces, rendering them stronger against minor trauma and less susceptible to HPV basal layer cell infection^6^.

Only 3% (3/100) of the subjects in our sample claimed to be smokers, a fact that certainly did not impact the results reported.

For alcohol consumption, 32% (32/100) of the subjects claimed they did not drink, 55% (55/100) said they had it once a week, 3% (3/100) had it twice a week, 6% (6/100) had it three times a week, and 4% (4/100) did not respond. Alcohol consumption was therefore reduced and of little significance. High alcohol consumption patterns seem to work synergistically with high risk HPV infection^39^, a factor not applicable to the studied sample.

Therefore, the impact of tobacco and alcohol consumption upon oral mucosa HPV infection should be further clarified.

Persistence of HPV infection and immune aspects: infection by HPV takes place at the beginning of sex life, somewhere between adolescence and early twenties. In most of the cases the infection is transient, and no clinical evidence indicates whether the disease can be suppressed or eliminated at all, unless immune incompetence sets in. Some persistent infections by high-risk virus types may evolve to genital cancer. Most of the time, the disease is diagnosed between the ages of 25 and 29 years^45^. There are evidences that HPV infection is a transient or intermittent phenomenon, with an average duration of 12 months^6^. There seems to be a difference in viral biologic activity between men and women, as male subjects tend to have more of a fluctuating infection with periods of spontaneous remission possibly due to their immune status, local factors, and the various forms of organization of the genital epithelium in both genders^13^. Our study looked into a narrowly aged population (mean age = 22.65 years) of both genders (60 females and 40 males), and samples may have been collected during a healing period.

Impact of diagnostic tool limitation: there still is variation in detection rates, depending on the chosen mucosal sample collection method, namely scrapes, lavages, or biopsy and the elected PCR assay^46^. Although scrapes were used and samples were kept refrigerated so as to allow for better detection of HPV DNA^47^,^48^, this method extracts only superficial epithelial cells that are infected in subclinical and clinical infection. Therefore, no viruses from latent infections in the basal or suprabasal layer cells are removed^29^. Primers MY09 and MY11 and GP5+/GP6+ were used in our study, as they are the most routinely used in HPV DNA detection and are able to identify a wide range of different viruses in one single reaction^7^,^49^. Reports indicate differences in the results obtained with different primers for one same sample, meaning different primers amplify different HPV genotypes. Detection of mixed infections can only be done by using various primer pairs in the PCR test^50^. Considering this limitation, not using various primer pairs in our study would increase the number of false negative test results. The wide variation in prevalence rates observed in various papers may be partially explained by the difference found in biopsies of normal patients and subjects with lesions in different tests (SB and PCR)^19^. Different tests done on one same group of samples could add consistency to comparative analyses. Another similar study, however enrolling fewer subjects, found positive HPV PCR tests in three patients, while the SB of these same patients was negative for HPV^17^. This reinforces the fact that results will depend on the sensitivity of each test.

The existence of local factors, as well as the fact that saliva has components that may have a protective effect such as lysozymes, lactoferrin, IgA, and cytokines^13^ could explain the low transmission rates through self-inoculation and oral sex^7^.

Although the fact that virus prevalence rates range between 0% and 100% in normal individuals indicates the possibility that the oral mucosa may work as a reservoir of viruses^19^,^34^, HPV may be considered as a risk factor for oral cancer. Immune disorders could impact HPV infection towards latency, cure, or carcinogenesis. HPV types 16 and 18 - associated with early proteins (E6 and E7) which inhibit tumor suppressing genes (p53 and pRB)1 - induce increased rates of mitosis, thus enhancing the possibility of viral DNA being integrated to cell DNA. Integrated HPV DNA found in SCC genome points to the thesis that this virus could be an important factor in the etiology of some head and neck carcinomas^21^,^22^.

## CONCLUSION

No traces of HPV were found in our sample based on the tests performed in our study. The vast variation (0–100%) on HPV prevalence rates found in the oral mucosa of healthy individuals found in the literature may indicate that the methods used to detect the virus need to be reassessed. It is our belief that more studies should be conducted to assist in the development of new methods that introduce new elements to clarify how HPV behaves in oral mucosa infection.

## References

[1] Oliveira MC, Soares RC, Pinto LP, Costa ALL. (2003). HPV e carcinogênese oral: revisão bibliográfica. Rev Bras Otorrinolaringol.

[2] Castro TMPPG, Bussoloti Filho I (2006). Prevalência do papilomavírus humano (HPV) na cavidade oral e na orofaringe. Braz J Otorhinolaryngol.

[3] Collier B, Goodbar-Larson L, Sokolowski, Schwartz S. (1998). Translationl Inhibition in Vitro of Human Papillomavirus Type 16 L2 mRNA Mediated trough Interaction with Heterogenous Ribonucleoprotein K and Poly (rC)- binding Proteins 1 and 2. J Biol Chem.

[4] Baker TS, Newcomb WW, Olson NH, Cowsert LM, Olson C, Brown JC (1991). Structures of bovine and human papillomaviruses. Biophys J..

[5] Swygart C. (1997). Human papillomavirus: disease and laboratory diagnosis. Br J Biomed Sci..

[6] Sinogas C, Rodrigues A, Reis D. Papilomavírus Humano Biologia e Epidemiologia. Universidade de Évora, Departamento de Biologia. http://evunix.uevora.pt/~sinogas/TRABALHOS/2004/papiloma.htm

[7] Castro TMPPG. (2007). Tese (Doutorado).

[8] Garlick JÁ, Taichman LB (1991). Human papillomavirus infection of the oral mucosa. Am J Dermatopathol.

[9] Summersgill KF, Smith EM, Levy BT, Allen JM, Haugen TH, Turek LP (2001). Human papillomavirus in the oral cavities of children and adolescents. Oral Surg Oral Med Oral Pathol.

[10] Hou SY, Wu SY, Chieng C. (2002). Transcriptional activity among high and low risk human papillomavirus E2 proteins correlates with DNA binding. J Biol Chem.

[11] Tinoco JA, Silva AF, Oliveira CAB, Rapoport A, Fava AS, Souza RP (2004). Correlação da infecção viral pelo papilomavírus humano com as le-sões papilomatosas e o carcinoma epidermoide na boca e orofaringe. Rev Assoc Med Bras.

[12] Ozbun MA (2002). Infectious human papillomavirus type 31b: purification and infection of an immortalized human keratinocyte cell line. J Gen Virol.

[13] Xavier SD (2007). Frequência de aparecimento do papilomavírus humano (HPV) na mucosa oral de homens com HPV genital confirmado por biologia molecular.

[14] Zur Hausen, de Villiers EM (1994). Human Papillomaviruses. Annu Rev Microbiol.

[15] Gutman LT, Herman-Giddens ME, Phelps WC (1993). Transmission of human genital papillomavirus disease: comparison of data from adults and children. Pediatrics.

[16] Cason J, Kaye JN, Jewers RJ, Kambo PK, Bible JM, Kell B (1995). Perinatal infection and persistence of human papillomavirus types 16 and 18 in infants. J Med Virol.

[17] Tominaga S, Fukushima K, Nishizaki K, Watanabe S, Masuda Y, Ogura H. (1996). Presence of human papillomavirus type 6f in tonsilar condiloma acuminatum and clinically normal tonsillar mucosa. Jpn J Clin Oncol.

[19] Kellokoski JK, Sirjänen SM, Chang F, Yliskoski M, Syrjänen KJ (1992). Southern blot hybridization and PCR in detection of oral human papillomavirus (HPV) infections in women with genital HPV infections. J Oral Pathol Med..

[20] Soares CP, Malavasi I, Reis RI, Neves KA, Zuanon JAS, Benatti Neto C, Spolidório LC, Oliveira MRB. (2002). Presença de papilomavírus humano em lesões malignas de mucosa oral. Rev Soc Bras de Med Trop.

[21] Dekmezian RH, Batsakis JG, Goepfert H. (1987). In situ hibridization of papillomavirus DNA in head and neck squamous cell carcinomas. Arch Otolaryngol Head Neck Surg..

[22] Alvarez IA, Lazo PS, Gonzalez SR, Tapia PR, Batalla FN, Nieto CS (1997). Using polymerase chain reaction to human papillomavirus in oral and pharyngolaryngeal carcinomas. Am J Otolaryngol.

[23] Giuliano AR, Siegel EM, Roe DJ, Ferreira S, Baggio ML, Galan L. (2003). Dietary Intake and Risk of Persistence Human Papillomavirus (HPV) Infection: The Ludwig-McGill HPV Natural History Study. J Infect Dis.

[24] Piyathilake CJ, Henao OL, Macaluso M, Cornwell PE, Meleth S, Heimburger DC (2004). Folate is associated with the natural history of high-risk human papillomaviruses. Cancer Res..

[25] Maitland NJ, Cox MF, Lynas C, Prime SS, Meanwell CA, Scully C. (1987). Detection of human papillomavirus DNA in biopsies of human oral tissue. Br J Cancer.

[26] Scully C, Maitland NJ, Cox MF, Prime SS (1987). Human papillomavirus DNA and oral mucosa. Lancet.

[27] Jenison AS, Xiu-ping Y, Valentine J, Koutsky LA, Christiansen AE, Beckman AM (1990). Evidence of prevalent genital-type human papillomavirus infections in adults and children. J Infect Dis.

[28] Yeudall WA, Campo MS (1991). Human papillomavirus DNA in biopsies of oral tissues. J Gen Virol.

[29] Jalal H, Sandres CM, Prime SS, Scully C, Maitland NJ (1992). Detection of human papilloma vírus type 16 DNA in oral squames from normal young adults. J Oral Pathol Med..

[30] Cruz IBF, Snijders PJF, Steenbergen RDM, Meijer CJLM, Snow GB, Walboomers JMM. (1996). Age dependence of human papilloma vírus DNA presence in oral squamous cell carcinomas. Oral Oncol Eur J Cancer.

[31] Badaracco G, Venuci A, Leonardo AD, Scambia G, Mozzetti S, Panici PB (1998). Concurrent HPV infection in oral and genital mucosa. J Oral Pathol Med..

[32] Schwartz SM, Daling JR, Doody DR, Wipf GC, Carter JJ, Madeleine MM (1998). Oral Cancer Risk in Relation to Sexual History and Evidence of Human Papillomavirus Infection. J Natl Cancer Inst.

[33] Smith EM, Hoffman HT, Summersgill KS, Kirchner HL, Turek L, Haugen TH (1998). Human papillomavirus and risk of oral cancer. Laryngoscope.

[34] Terai M, Hashimoto K, Yoda K, Sata T. (1999). High prevalence of human papillomaviruses in the normal oral cavity of adults. Oral Microbiol Immunol.

[35] Creatsas G. (1995). Sequelae of premature sexual life. J R Soc Med..

[36] Cañadas MP, Bosh FX, Junqueira ML, Ejarque M, Font R, Ordoñez E, Sanjosé S. (2004). Concordance of prevalence of human papillomavirus DNA in anogenital and oral infections in a high risk population. J Clin Microbiol.

[37] Centurioni MG, Puppo A, Merlo DF, Pasciucco G, Cusimano ER, Sirito R (2005). Prevalence of human papillomavirus cervical infection in an Italian asymptomatic population. BMC Infect Dis.

[38] Minkoff H, Feldman JG, Strickler HD, Watts DH, Bacon MC, Levine A (2004). Relationship between Smoking and Human Papillomavirus Infections in HIV-Infected and -Uninfected Women. J Infect Dis.

[39] Smith EM, Ritchie JM, Summersgill KF, Hoffman HT, Wang DH, Haugen TH (2004). Human Papillomavirus in Oral Exfoliated Cells and Risk of Head and Neck Cancer. J Natl Cancer Inst.

[40] Gárcia-Milián G, Hernández H, Panadé L, Rodríguez C, González N, Valenzuela C (1998). Detection and typing of human papillomavirus DNA in benign and malignant tumors of laryngeal epithelium. Acta Otolaryngol (Stockh).

[41] Herrero R, Castellsagué X, Pawlita M, Lissowska J, Kee F, Bala-ram P. (2003). Human Papillomavirus and Oral Cancer: The International Agency for Research on Cancer Multicenter Study. J Natl Cancer Inst.

[42] Dahlstrom KR, Adler-Storthz K, Etzel CJ, Liu Z, Dillon L, El-Naggar AK, Spits MR (2003). Human Papillomavirus Type 16 Infection and Squamous Cell Carcinoma of the Head and Neck in Never-Smokers. Clin Cancer Res..

[43] Syrjänen S. (2004). HPV infections and tonsillar carcinoma. J Clin Pathol.

[44] Ritchie JM, Smith EM, Summersgill KF, Hoffman HT, Wang D, Klus-smann JP (2003). Human papillomavirus infection as a prognostic factor in carcinomas of the oral cavity and oropharynx. Int J Cancer.

[45] Russomano F, 2000. Presença de HPV nos fluidos em geral. Available from: URL: http://www.cervical.com.br.

[46] Ha PK, Califano JA (2004). The role of human papillomavirus in oral carcinogenesis. Crit Rev Oral Biol Med..

[47] Hoffmann M, Khan T, Mahnke CG, Goeroegh T, Lippert BM, Werner JA (1998). Prevalence of human papillomavirus in squamous cell carcinoma of the head and neck determined by polymerase chain reaction and southern blot hybridization: proposal for optimized diagnostic requirements. Acta Otolaryngol (Stockh).

[48] Melo A, Roa I, Montenegro S, Capurro I, Roa JC (2005). Estúdio comparativo de detección del vírus papiloma humano (VPH) en muestras citoló-gicas y biopsias de cuello uterino. Rev Med Chile.

[49] Ribeiro KMX. (2002). Estudo da ocorrência do papilomavírus humano em tonsilas palatinas na população pediátrica.

[50] Kado S, Kawamata Y, Shino Y, Kasai T, Kubota K, Iwasaki H (2001). Detection of human papillomavirus in cervical neoplasias using multiple sets of generic polymerase chain reaction primers. Gynecol Oncol.

